# Nonlinear Analysis of Eye-Tracking Information for Motor Imagery Assessments

**DOI:** 10.3389/fnins.2019.01431

**Published:** 2020-01-15

**Authors:** Antonio Lanata, Laura Sebastiani, Francesco Di Gruttola, Stefano Di Modica, Enzo Pasquale Scilingo, Alberto Greco

**Affiliations:** ^1^Department of Information Engineering & Research Centre E. Piaggio, School of Engineering, University of Pisa, Pisa, Italy; ^2^Department of Translational Research and New Technologies in Medicine and Surgery, University of Pisa, Pisa, Italy

**Keywords:** motor imagery, eye-tracking, phase space, recurrence quantification analysis, mental chronometry

## Abstract

This study investigates the assessment of motor imagery (MI) ability in humans. Commonly, MI ability is measured through two methodologies: a self-administered questionnaire (MIQ-3) and the mental chronometry (MC), which measures the temporal discrepancy between the actual and the imagined motor tasks. However, both measures rely on subjects' self-assessment and do not use physiological measures. In this study, we propose a novel set of features extracted from the nonlinear dynamics of the eye gaze signal to discriminate between good and bad imagers. To this aim, we designed an experiment where twenty volunteers, categorized as good or bad imagers according to MC, performed three tasks: a motor task (MT), a visual Imagery task (VI), and a kinaesthetic Imagery task (KI). Throughout the experiment, the subjects' eye gaze was continuously monitored using an eye-tracking system. Eye gaze time series were analyzed through recurrence quantification analysis of the reconstructed phase space and compared between the two groups. Statistical results have shown how nonlinear eye behavior can express an inner dynamics of imagery mental process and may be used as a more objective and physiological-based measure of MI ability.

## 1. Introduction

Motor imagery (MI) is a cognitive process by which an individual rehearses or simulates a given action (Choudhury et al., 2007). Many studies have provided evidence on the positive effects of MI in both healthy subjects and patients (Dickstein and Deutsch, 2007; Tong et al., 2017). Indeed, MI can improve basic motor skills and sport performance and can offer a beneficial, non-invasive support to standard rehabilitation therapies (Decety and Ingvar, 1990; Driskell et al., 1994; Butler and Page, 2006; Sharma et al., 2006; De Vries and Mulder, 2007; Guillot and Collet, 2008; Di Rienzo et al., 2014). Moreover, it has been successfully applied to treat chronic pain (i.e., complex regional pain syndrome, phantom limb pain, and back pain (Bowering et al., 2013).

To date, MI ability is commonly measured through two main methodologies: self-administered questionnaires and mental chronometry (MC) (Moran et al., 2012). A widely used questionnaire is the Motor Imagery Questionnaire-3 (MIQ-3), which is a self-reported assessment of the ability to recreate a mental motor representation (Williams et al., 2012). The MC is the measure of the temporal discrepancy between the actual and the imagined motor tasks (Moran et al., 2012; Williams et al., 2015). This approach grounds on the fact that executed and imagined tasks show overlapped neural patterns, comparable psychophysiological profiles, and similar temporal duration (Guillot et al., 2010a). Thus, MC provides semi-quantitative information about the temporal congruence between executed and imagined movements (Guillot et al., 2010b) and it is considered an objective measure of MI ability (Collet et al., 2011), even if the exact timing (onset and offset) of the imagination process is, actually, self-reported. In the literature, MC has been applied in several fields such as cognitive psychophysiology (Heremans et al., 2008), cognitive neuroscience (Spruijt et al., 2013), and behavioral neuroscience (Bakker et al., 2007).

Due to the dependency of both methods on the subjective interpretation of the mental process none of them does actually provide an objective measure of the inter-individual physiological differences underlying MI abilities (Isaac, 1992; Roure et al., 1999; Miller and Saygin, 2013; Sakurada et al., 2016; Saruco et al., 2017). Hence, a reliable measure of MI ability would be crucial to correctly assess these inter-individual differences.

A further variable to be considered in the MI field is the sensory modality (kinaesthetic, visual) and the perspective from which the imagery task is executed (first-person, third-person) (Williams et al., 2012). In cognitive neuroscience, most of the researchers consider motor imagery as a first-person process, i.e., the mental representation of one's self-performing a motor action without any overt movement (Moran et al., 2012; Filgueiras et al., 2017). Previous studies have investigated possible relationships between the two metrics (i.e., MIQ-3 and MC) for both kinaesthetic and visual imagery modalities. However, no significant correlations have been reported so far (Lequerica et al., 2002). In addition, they have noted that MIQ-3 scores significantly differed between visual and kinaesthetic tasks, while the MC did not. These findings have suggested that MIQ-3 and MC address different properties of imaginary ability and they could be considered together as part of a comprehensive assessment of MI (Collet et al., 2011; Moran et al., 2012; Williams et al., 2015).

The aforementioned limitations have led recent studies to propose physiologically-based methods for a more objective assessment of MI ability. Particularly, they have mostly used brain activity information to measure the real engagement of an individual in a MI task as well as the goodness of the mental representation (Popivanov et al., 2006; Soe and Nakagawa, 2008; Andrade et al., 2014; Baravalle et al., 2018; Pavlov et al., 2018, 2019; Catrambone et al., 2019).

In this context, the eye-movement dynamics provides interesting prospective. In fact, previous studies have investigated eye-gaze dynamics as a reliable measure of MI in a variety of motor tasks (Mast and Kosslyn, 2002; Gueugneau et al., 2008; Heremans et al., 2008) and, more recently, the combination of eye-gaze and brain dynamics have been used for new hybrid brain computer interfaces (Meena et al., 2015; Wang et al., 2015). These studies have shown that eye movements support the process of image generation during visual imagery and that this is not an epiphenomenon (Andrade et al., 1997, 2014; Lanata et al., 2015). These findings have suggested that participants use memories of eye movements to help recreating mental images (Mast and Kosslyn, 2002). Particularly, Laeng et al. (Laeng and Teodorescu, 2002) have demonstrated that not only eye movements are constantly present during mental imagery but, more specifically, they are functionally involved in mental imagery processes. Indeed, they have found that during imagery processes eye movements reflect the conditions in which the participants have studied the stimulus to be imagined. In case the participant maintains fixation while studying the stimulus, there were almost no eye movements during the imagery phase. Vice-versa, in case the subject visually explored the stimulus, he/she also moved his/her eyes during the imagery phase. Furthermore, the sequence of fixation during imagery and perception was very similar. In fact, the more similar the imagery and perception scan paths, the better the participants performed in a memory task (Brandt and Stark, 1997; Laeng and Teodorescu, 2002). A further step has been taken by de'Sperati (2003), when he has demonstrated that eye movements could be used as markers of the spatio-temporal evolution of mental imagery processes.

Therefore, assuming the eye-gaze as the output of a time-variant dynamical system, the study of its time evolution, i.e., the eye movement dynamics, could provide an easy and robust indication of the quality of cognitive process underlying the motor imagery. Moreover, it is worthwhile noting that, as for many physiological phenomenon, recently, a chaotic behavior of eye movement dynamics has been shown in healthy humans (Aştefănoaei et al., 2013). For example, relevant information has been found on saccadic eye movements using a semi-quantitative approach through indexes derived from chaos theory such as fractal dimension and largest Lyapunov exponent (Poiroux et al., 2015; Frank et al., 2016).

In sight of this, we conducted an experiment to demonstrate whether nonlinear eye dynamics could be used to distinguish between good and bad motor imagination performance. Among the large number of nonlinear methods, we do believe Recurrence Quantification Analysis (RQA) is an effective way to extract information from the eye movement dynamics. Indeed, RQA has been previously applied to describe the temporal dynamics of eye movements during picture presentation (Anderson et al., 2013; Vaidyanathan et al., 2014; Farnand et al., 2016), and to study the temporal organization of eye movements during the mental imagery of previously observed pictures. Particularly, high percentage of recurrent fixations and determinism values have been reported during mental imagery. These findings have been assumed to reflect the visuospatial working memory processes by which mental images are generated and maintained in mind (Gurtner et al., 2019). Moreover, the large inter-individual differences found in RQA measures during mental imagery has been related to the individual differences in working memory ability (Gurtner et al., 2019). Based on these previous studies, we have assumed that RQA could be used to find effective markers of motor imagery ability as well as of motor imagery modalities.

To this aim, we have investigated the temporal dynamics of the eye movements, through RQA of the reconstructed phase space, which allows identifying complex and nonlinear eye gaze behavior (Eckmann et al., 1987; Casdagli, 1997; Marwan et al., 2002, 2007). In this study we statistically compared RQA features between subjects labeled as good and bad imager according to their MC in order to propose a more objective and physiological-based measure of MI ability.

## 2. Methods

### 2.1. Subjects Recruitment and Acquisition Set-Up

The study was performed in accordance with the ethical standards of the Declaration of Helsinki. We enrolled 20 volunteers (9 females; mean age = 25, range = 20–30) from a pool of students of the University of Pisa. All involved participants had no history of medical or neurological disorders and reported normal/corrected-to-normal vision. The study was approved by the Bioethics Committee of the University of Pisa. Before starting the experimental procedure participants were asked to answer some questionnaires to evaluate the handedness (Oldfield, 1971), the ability to imagine motor actions (MIQ-3, Williams et al., 2012), the level of subjectively perceived anxiety (STAI-Y, Julian, 2011), and the mood (PANAS, Watson et al., 1988). Participants whose scores of PANAS and STAY were not within the normative ranges [e.g., STAY-Y scores > 45, and PANAS: Negative affect NA>30 (>95th percentile) e Positive affect PA <18 (< 5th percentile)], thus indicating the possible presence of affective disorders, were not included in the study.

**Figure 1:**
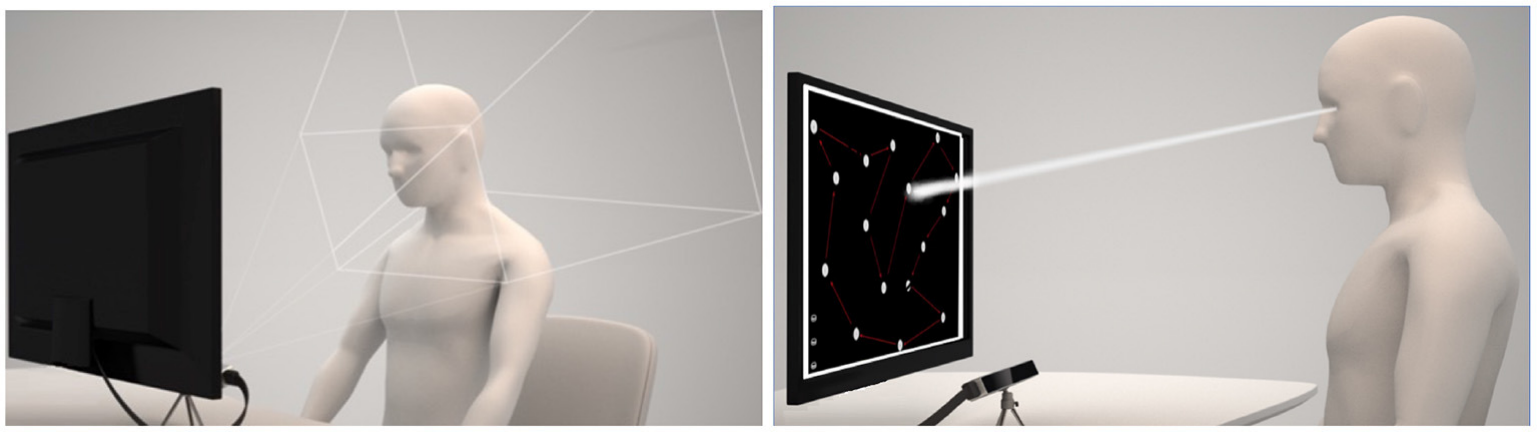
Experimental Set-up: The picture on the left emphasizes the area in which the eye tracker can correctly acquire subject's eyes. The picture on the right shows the eye tracker position and computed gaze direction of subject while performing the imagery task.

All experiments were performed in the same room at the University of Pisa, with controlled illumination and in the same daytime interval (11:00–16:00). Each participant was asked to sit on a comfortable chair in front of a desk, on which was placed a tablet and the eye tracker system. Throughout the whole experiment the eye-gaze was continuously monitored by means of Eye Tribe remote eye-tracker system (ET, The EyeTribe 2014). The experiment was App-guided, and the app was developed in Visual Studio 2017 in *C#* language (see Figure 1).

After the experiment, participants have been clustered into two groups according to the MC score distribution (see section 3): good imagers (GI) (MC < Median_*MC*_) and bad imagers (BI) (MC ≥ Median_*MC*_).

### 2.2. Experimental Protocol and Subject Clustering

**Figure 2:**
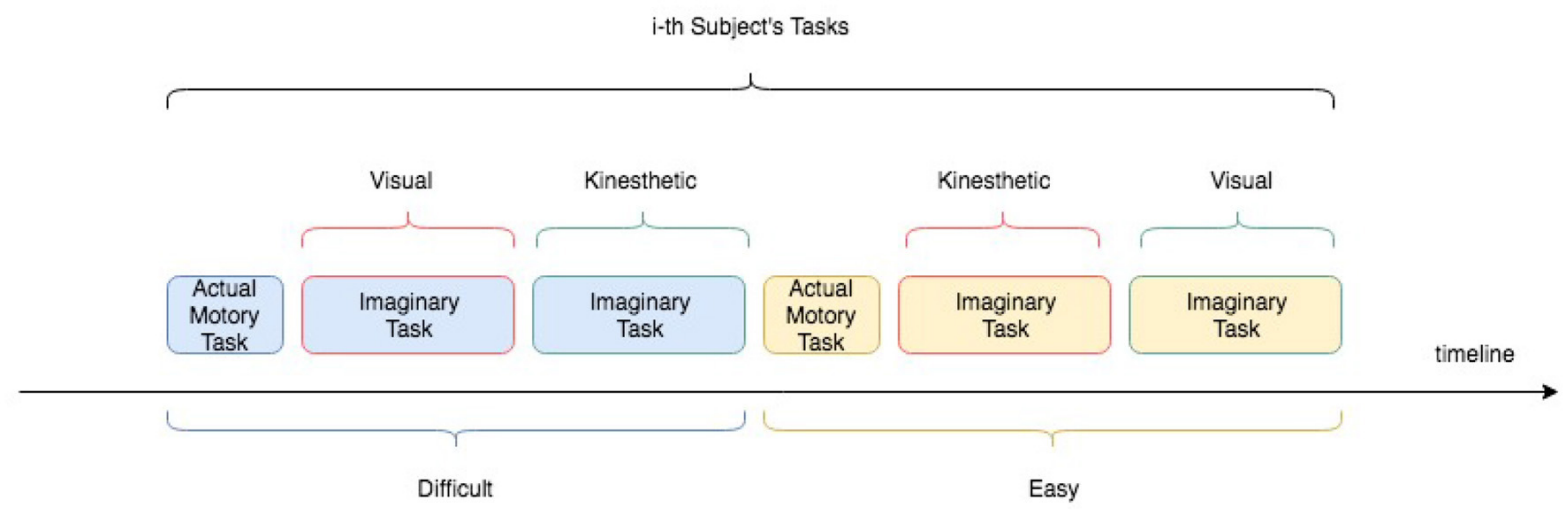
Experimental protocol timeline for an i-th subject. Easy and Difficult options as well as Visual and kinaesthetic Imagery tasks were counterbalanced among the subjects.

**Figure 3:**
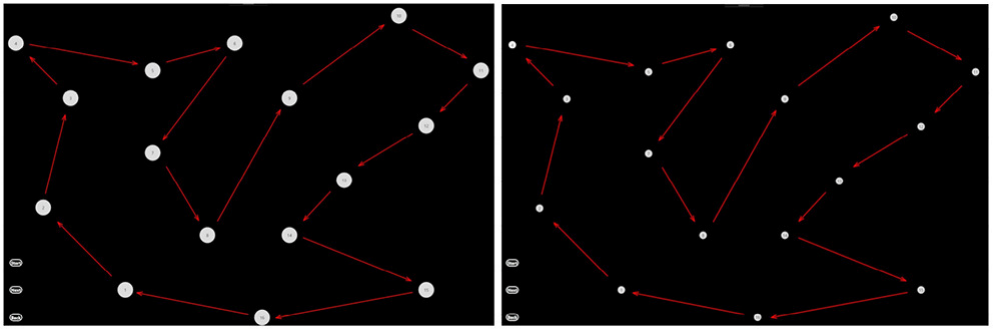
Interactive interface used for motor imagery assessment. **(Left)** The easy task; **(Right)** the difficult task.

**Figure 4:**
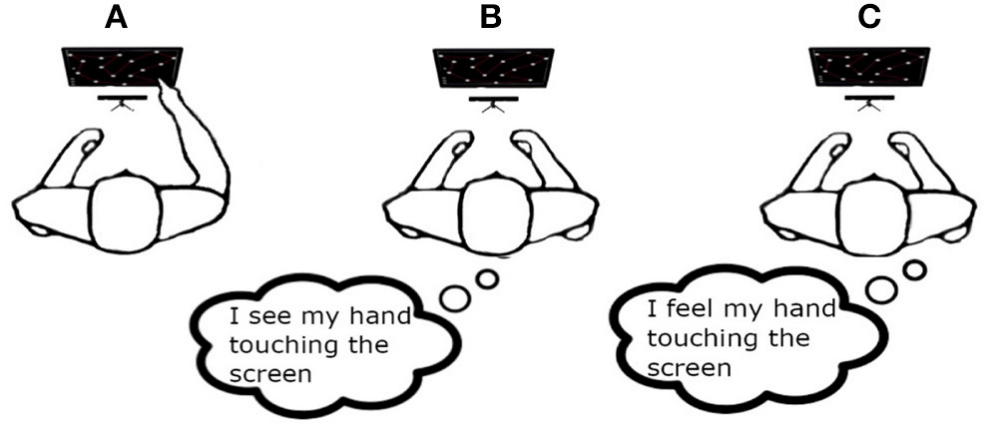
The Figure shows the three experimental sessions: **(A)** motor task (MT); **(B)** visual imagery (VI); **(C)** kinaesthetic imagery (KI).

The experiment consisted of acting and imaging different visuomotor tasks following the timeline showed in the Figure 2. More specifically, each participant was asked to interact with a software application, which guided the subjects to press or imagine to press a sequence of “buttons” on a touch-screen of a tablet and following a specific path indicated by the red arrows (see Figure 3). Two variants of the protocol were proposed based on the size of the buttons: an “easy” option (big buttons) and a “difficult” option (small buttons) (see Figure 3). Each participant performed both options in a randomized order. For each option, the experiment timeline consisted of a Motor task (MT), a Visual Imagery task (VI), and kinaesthetic Imagery task (KI), as follows (see Figure 4):

- Motor task (MT): the subject performed the motor task by pressing the buttons in the right sequence.
- Visual Imagery task (VI): the subject was asked to imagine himself/herself while performing the motor task described above observing the scene from an internal perspective, i.e., seeing his/her hand touching the screen.
- Kinaesthetic Imagery task (KI): the subject is asked to image himself/herself while performing the motor task in “first person”, i.e., paying attention to the information coming from his/her body parts: the same sensations he/she would feel while is performing a real motor task.

Participants were asked to say “START” and “STOP”, respectively, at the beginning and the end of each imagery task. The timing of each task was measured by an experimenter with a chronometer. The two imagery tasks (i.e., VI and KI) always came after the motor one, but the number of the VI and KI tasks was counterbalanced among participants.

At the end of each imagery task, the imagery performance was evaluated in two ways:

- By filling out a slightly revised version of the MIQ-3 questionnaire, which consists of a 12-item self-report inventory evaluating the individual ability to shape mental images of motor actions. For each item the subject had to rate the difficulty of the performed imagination task by using a 7-point scale (from 1 = very hard to see/feel; 4 = neutral (not easy/not hard); 7 = very easy to see/feel, and intermediate levels). The final score is the average of the ratings obtained in the four MI tasks.
- By computing the MC as the absolute difference between time of execution of the motor tasks and the time of execution of the imagined tasks. Specifically, MC shows the discrepancy between execution time as follows: $\text{MC}=\left|\left(T_{A}-T_{I}\right)\right|$, where *T*_*A*_ and *T*_*I*_ are the execution time of the motor and imaginary task, respectively.

### 2.3. Signal Processing Method for Eye-Tracking Feature Extraction

In the following sections, we describe the methodology applied to eye gaze time series to characterize the eye gaze nonlinear dynamics. Particularly, we explain the procedure to reconstruct the dynamical system state space from which a set of parameters are computed: the integration of the bi-dimensional information of the gaze point; the reconstruction of the embedded phase space and the extraction of the RQA nonlinear-complexity features; and the calculation of the time standard eye-tracking measures, such as fixation time and number of blinks.

#### 2.3.1. Point-to-Point Instantaneous Gaze Direction

Point-to-Point Instantaneous Gaze Direction (PPIGD) (i.e., the angle θ_*i*_) was computed as the angle between the vector obtained by two consecutive points of gaze and the horizontal axis. This signal was the result of the integration of the bi-dimensional gaze point into a mono-dimensional time series. More in detail, given the *i*_*th*_ gaze point $GP_{i}\equiv \left(\begin{array}{c} x\\ y \end{array}\right)$, we can define the PPIGD as follows (Aks et al., 2002):

(1) $$\begin{array}{l} \theta_{i}=\arctan\frac{\Delta GP_{y,i}}{\Delta GP_{x,i}} \end{array}$$

where $\Delta GP_{i}\left(\begin{array}{c} x\\ y \end{array}\right)=GP_{i+1}\left(\begin{array}{c} x\\ y \end{array}\right)-GP_{i}\left(\begin{array}{c} x\\ y \end{array}\right),\Delta GP_{x,i}=(GP_{x,i+1},GP_{x,i}),\Delta GP_{y,i}=(GP_{y,i+1},GP_{y,i})$.

#### 2.3.2. Phase-Space (PS) and Phase Space Reconstruction

The Phase space (PS) was reconstructed starting from the PPIGD time series. PS allows the representation of dynamical system through a time-evolution law. As a matter of fact, each element of the PS represents a possible state of the system (Marwan et al., 2007). Hence, knowing the time-evolution law, once a present state is fixed, all of the future states are determined as well (Lajish et al., 2012). This means that a point in PS specifies the state of the system and vice versa. Therefore, we could investigate the dynamics of the system by studying the dynamics of the corresponding PS points (Piotrowski et al., 2004). Since in many cases this dynamics is yet to be known, we can obtain an equivalent dynamics reconstructing a PS by using the Takens's theorem (Takens, 1981). This guarantees that the PS geometrical properties of a given nonlinear system can be reconstructed by using copies of the times series measured, as the output of the original system. The reconstructed PS is representative of the dynamics of the original system, moreover, it is a vector space (Kantz and Schreiber, 2004) in which, by using a *time* *delay* *embedding* method, we can describe the system dynamics by an *m*−*dimensional* map. In the univariate case, it is represented by the following embedding vector:

(2) $$\begin{array}{l} {\text{x}}_{n}=\left(x_{n},x_{n-\tau },\ldots ,x_{n-\left(m-1\right)\tau }\right) \end{array}$$

where ${\left\{x_{n}\right\}}_{n=1}^{N}$, *n* = 1, …, *N*, is the measured time series. *m* is the embedding dimension, i.e., the number of components in *x*_*n*_, and τ is the time delay.

**Figure 5:**
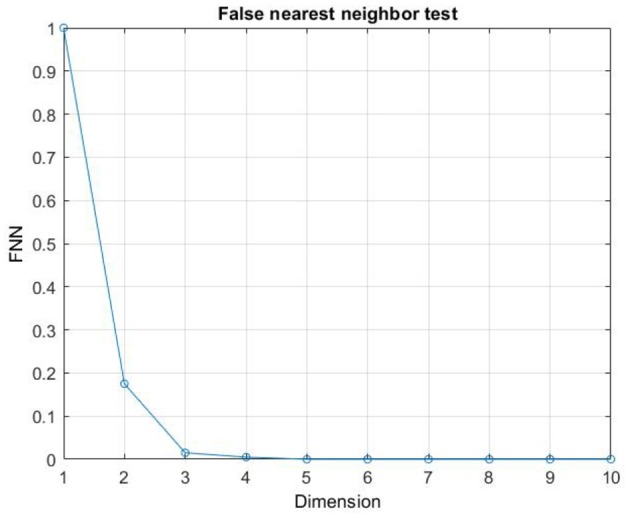
Example of the computation of the Embedding dimension *m*.

**Figure 6:**
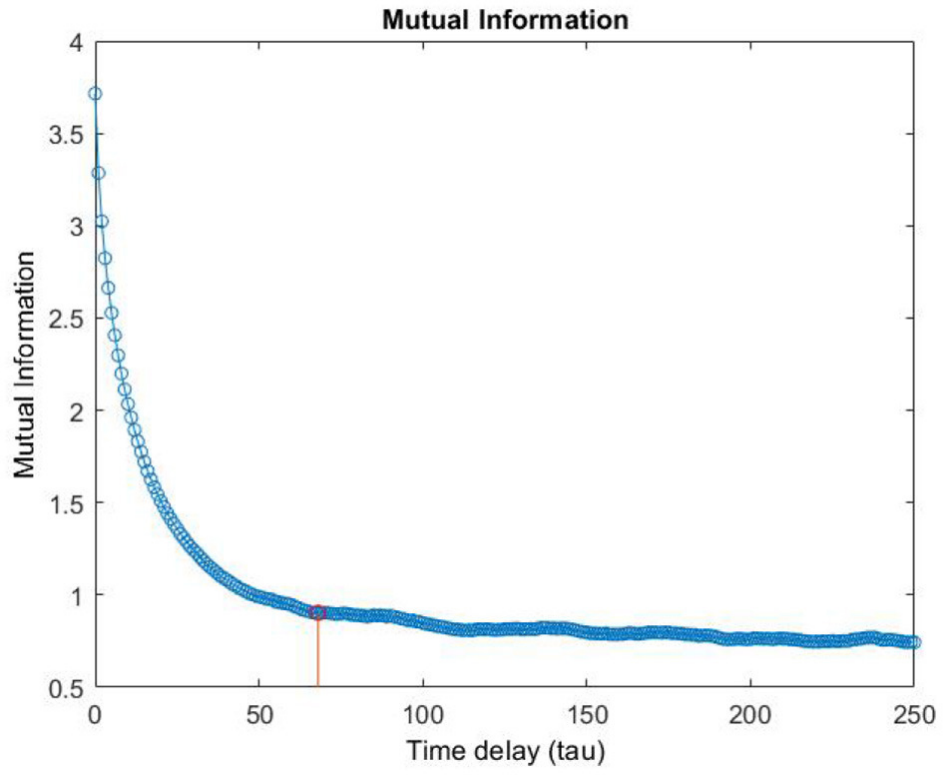
Example of the computation of the Time delay τ.

Although in the literature, many approaches have been proposed for the selection of *m* and τ (Fraser, 1986; Albano et al., 1987; Kennel et al., 1992; Kaplan, 1993; Chun-Hua and Xin-Bao, 2004), we computed embedding dimension, *m*, as the first minimum of the false nearest neighbors function over the possible dimensions from zero to ten. An embedding dimension of *m* = 4 was obtained (Stephen et al., 2009; Kraemer et al., 2018) (see Figure 5 as en example of the *m* computation). Furthermore, Time delay τ was computed as the first minimum of the mutual information profile, maximizing the independence among the components of the embedding vector (see Figure 6 as an example of the τ computation). Finally, the RQA was applied to the reconstructed phase space in order to quantify the dynamic of the eye evolution throughout the process.

#### 2.3.3. Recurrence Quantification Analysis

The RQA is a method for quantifying the dynamic properties of a system represented in the phase space (Webber and Zbilut, 1994; Marwan et al., 2007). RQA is based on the recurrence plot (RP), which visualizes recurrences of a state vector *x*_*i*_(*i* = 1, ..., *N*) in the phase space. Specifically, RP is a graph showing those instants during which a state of the dynamical system recurs, i.e., RP reveals all the time points when the phase space trajectory visits roughly the same area in the phase space.

(3) $$\begin{array}{l} {\text{R}}_{i,j}=\Theta \left(\epsilon -\|x_{i}-x_{j}\|\right),i,j=1,\ldots ,N, \end{array}$$

where *N* is the number of measured points *x*_*i*_, ϵ is a threshold distance, ‖*‖ is a norm, e.g., the Euclidean norm, and Θ(*x*) is the Heaviside function.

A crucial issue of RP is the choice of the threshold ϵ. Specifically, if we choose a too small ϵ, there may be almost no recurrence points and we cannot learn anything about the recurrence structure of the underlying system. On the other hand, if we choose a too large ϵ, almost all the points are neighbor of all the other points. To date no optimum values of ϵ are currently in the scientific literature, and ϵ is chosen following different rules of thumb (Mindlin and Gilmore, 1992; Zbilut et al., 2002). Here, we have customized the value of ϵ for each time series as reported in Dabiré et al. (1998).

Recurrences are the building blocks from which all other measures in RQA are constructed. To our aim, the following features were calculated (Webber and Zbilut, 2005): Recurrence rate (*REC*), Determinism (*DET*), Laminarity (*LAM*), and Entropy (*ENTR*).

- The REC is defined as a measure of the density of recurrence points in the RP. Specifically, considering ***x***_*i*_ the time series of one variable, for *m* variables we have ***x***_*i*_ = (*x*_1,*i*_, …, *x*_*m, i*_), with *i* = 1, …, *N*. We define the recurrence matrix, *N*×*N*, of element *R*_*ij*_ as follows:
  (4) $$R_{ij}=\{\begin{array}{l} 1,\text{ if d}(x_{\text{i}},x_{\text{j}})<\epsilon \\ 0,\text{ otherwise} \end{array}$$
  where *d* is the distance between ***x***_*i*_ and ***x***_*j*_.
  The recurrence exits when *R*_*ij*_ = 1 with *i* ≠ *j*, the total number of recurrences is $R=\overset{N-1}{\underset{i=1}{\sum }}\overset{N}{\underset{j=1+1}{\sum }}R_{ij}$.
  *REC*, is defined by the equation:
  (5) $$\begin{array}{l} REC=\frac{100}{N\left(N-1\right)}2R \end{array}$$
  REC corresponds to the correlation sum.
- The determinism (DET) is defined as the percentage of recurrence points which form diagonal lines:
  (6) $$\begin{array}{l} DET=\frac{\sum_{l=l_{min}^{D}}^{N}lP_{D}\left(l\right)}{\sum_{l=1}^{N}lP_{D}\left(l\right)}, \end{array}$$
  where *D* is defined as the set of diagonal lines; *P*_*D*_(*l*) as the histograms corresponding to number of lines of D with length $l>l_{min}^{D}$. DET can be interpreted as the probability that two closely evolving segments of the phase space trajectory will remain close for the next time step. This measure provides indications on the predictability of the dynamical system. Of note, in deterministic systems, time series are commonly characterized by repeated similar state evolution (corresponding to local predictability) and exhibit very simple regular structures, which, accordingly to the RP construction, are reflected in many long diagonal lines. On the other hand, chaotic systems can show a certain regularity, but with much more complex and denser features, whereas unpredictable random signals, such as the white noise, are characterized by sparse points in the RP.
- The Laminarity (LAM) is defined as the percentage of recurrence points which form vertical lines:
  (7) $$\begin{array}{l} LAM=\frac{\sum_{l=l_{min}^{V}}^{N}lP_{V}\left(l\right)}{\sum_{l=1}^{N}lP_{V}\left(l\right)} \end{array}$$
  where *V* is defined as the set of vertical lines; *P*_*V*_(*l*) as the histograms corresponding to number of lines of V with length $l>l_{min}^{V}$. LAM is a measure of the probability that a state will not change at the next time step (i.e., it remains within a range defined by ϵ). This measure estimates the amount of laminar phases in the system, and can be considered an indirect measure of the intermittency, i.e., the irregular alternation of phases of apparently periodic and chaotic dynamics (Dutt-Mazumder et al., 2018).
- The Entropy (ENTR) is defined as the Shannon entropy. Let's define *p*(*l*) as the probability that a diagonal line has exactly length *l* = *l*_*min*_. This can be estimated from the frequency distribution of the probability distribution of the diagonal line lengths:
  (8) $$\begin{array}{l} p\left(l\right)=\frac{P_{D}\left(l\right)}{\sum_{l=l_{min}}^{D}P_{D}\left(l\right)} \end{array}$$
  Hence,
  (9) $$\begin{array}{l} ENTR=-\overset{N}{\underset{l=l_{min}}{\sum }}p\left(l\right)\ln\;p\left(l\right) \end{array}$$
  ENTR refers to the Shannon entropy of the probability *p*(*l*) of finding a diagonal line of exactly length *l* in the RP. It reflects the complexity of the RP with respect to the diagonal lines. It is an indication of the complexity of the deterministic structure in the system. However, this entropy depends sensitively on the bin number and, thus, may differ for different realizations of the same process.

#### 2.3.4. Fixation Time and Blink Detection

In addition to the RQA estimated from the PPIGD series, we calculated the fixation time and number of blinks for each experimental session. Specifically, fixation time is the time among saccade movements needed to correctly project a detail into the fovea. Generally, this process lasts about hundreds of milliseconds. However, when someone has to interpret details of an image, the fixation time can vary over time due to the cognitive process related to visual attention. In this study, we calculated the fixation time as the number of consecutive video-frames in which the Point-of-Gaze fell within the same specific area of the screen (i.e., area of fixation) multiplied by the sampling time of the camera *t*_*c*_. Here, we have chosen an area of fixation of about 3*x*3 pixel (Armato et al., 2013; Lanata et al., 2013).

Moreover, within each experimental session and for each task, we calculated the total number of blinks. The number of blinks was obtained considering the computed pupil area and the module of the gaze vector. The gaze vector was composed by the center of axes and the (*x* and *y*) coordinates of the gaze point. The number of blinks was obtained as the number of times in which the gaze vector module together with the pupil area were zero. Of note, the eye tracker system gave an output (gaze point) of zero when the gaze point went out of the borders of the calibration plane, but since there could be artifacts, that took the eye gaze out of the calibration plane, we considered the set of gaze vector and pupil area. We thresholded these variables and only when both of them went over the threshold, this was counted as a blink.

### 2.4. Statistical Analysis

**Figure 7:**
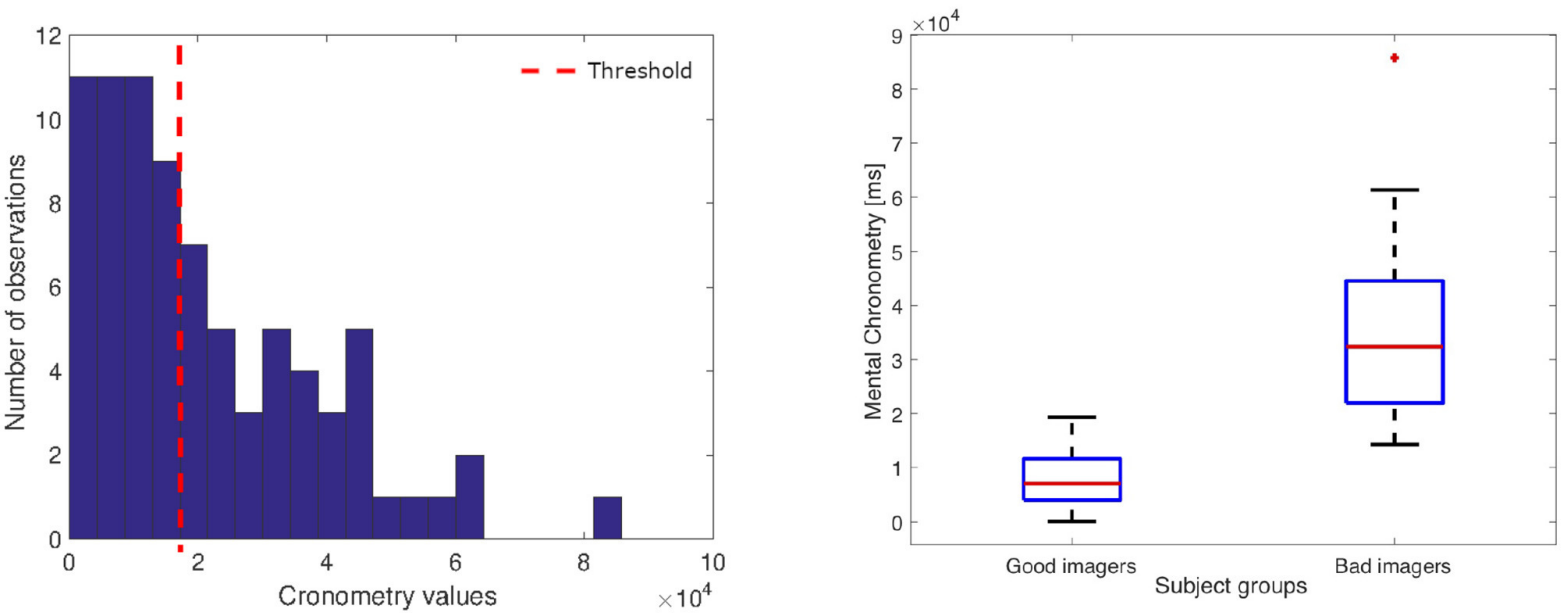
**(Left)** Histogram of the chronometry values; the dotted line indicates the threshold that split the good from bad imagers. **(Right)** Box-plot of the Good and Bad imagery MC distributions (GI: median: 7,078 ms, interquartile range (IQR): 7,651 ms; BI median: 32,380 ms, IQR: 22,574 ms).

As mentioned in section 2.2, each subjects' performance has been labeled as good (GI) or bad imagery (BI) according to the MC value. More specifically, we have calculated the median value of the distribution of the duration differences between the imagery tasks and the motor tasks (see Figure 7). Accordingly, each feature vector was associated with the GI group whether the MC was under or equal to the median threshold, or with the BI group in the other case.

Afterwards, the difference between these two groups have been statistically evaluated in terms of eye-gaze dynamics (fixation time, blink number, and RQA measures). Specifically, we performed the following statistical comparisons:

- Comparison between BI and GI considering both easy and difficult tasks together;
- Comparison between BI and GI considering only the easy task;
- Comparison between BI and GI considering only the difficult task.

To this aim, we have used a non-parametric Mann–Whitney *U*-test under the null hypothesis that the medians of the two groups were equal. Indeed, the Shapiro–Wilk test demonstrated that most of the features showed a non-Gaussian distribution (*p* < 0.05). All *p*-values were corrected following the Holm-Bonferroni's method. This addresses the problem of multiple statistical testing, which leads to a higher probability of a Type I error (probability of false positive). The Holm-Bonferroni method controls the family-wise error rate by adjusting the rejection criteria of each of the individual hypotheses. This method is less conservative than the classical Bonferroni method, reducing the related increase of type II error risk than this latter. In practice, the *p*-values are first sorted and then the smallest value is multiplied by N, where N is the number of comparisons. The next value is then multiplied by N-1 etc. Accordingly, the highest *p*-value remains unchanged (i.e., it is multiplied by 1). The corrected *p*-values are finally compared to the alpha level of 0.05.

Furthermore, we have performed also a comparison between the eye gaze features computed during the easy and the difficult tasks and between the kinaesthetic and the visual tasks. In this case, due to the paired nature of the data, we have adopted the Wilcoxon signed-rank test, which is a non-parametric test for paired data. Also in this case, the significance level has been set to 0.05 (5%) and all p-values have been corrected following the Holm-Bonferroni's method.

## 3. Results

In this section, we present the results of the statistical analyses described in section 2.4. Figure 7 shows the distribution of the MC values calculated for all tasks. The red line indicates the median value used to associate each performance with the BI and GI group. As expected, the histogram shows a skewed shape, with the peak close to the zero, which indicates that all good imagery performance obtained a similar, very small MC value.

In Tables 1–5, for all statistical comparisons, we report the *p*-value and median values (± median absolute deviation) of the RQA features, whereas blink number and fixation time are not reported since they did not yield any significant results. Of note, Tables show the *p*-value in bold when the difference between the two group is statistically significant.

**Table 1:** Statistical comparison between easy task (ET) and difficult tasks (DT).

**Feature**	***p*-value**	**ET Median ± MAD**	**DT Median ± MAD**
REC	0.6001	0.2612 ± 0.0137	0.2658 ± 0.0139
DET	0.4762	0.4230 ± 0.0206	0.4285 ± 0.0172
LAM	0.8507	2.3655 ± 0.0503	2.3766 ± 0.0462
ENTR	0.8507	0.4889 ± 0.0310	0.4941 ± 0.0226

Surprisingly, easy and difficult tasks did not show significant differences for all features (see Table 1). Instead, kinaesthetic and visual modalities revealed significant differences in the nonlinear domain features, i.e., REC, DET, and ENTR (Table 2). Table 3 shows the results of the statistical comparison between GI and BI for each of the RQA features. The comparison included both the values extracted from the difficult and the easy task together (i.e., considering the easy and difficult tasks as two repetitions of the same task). Each RQA feature showed a significant higher values for the BI group compared to the GI one, i.e., a higher complexity of the eye dynamics evaluated through the PPIGD time series. Interestingly, when we considered only the easy tasks, these statistical difference faded (Table 4), whereas they were still significant analyzing the difficult tasks, except for the REC (Table 5). Of note, dividing the feature-set according to the difficulty level of tasks, we also reduced the sample size and consequently, the *p*-values tended to be higher. This could explain the loss of significance for the REC parameter for the difficult tasks.

**Table 2:** Statistical comparison between kinaesthetic task (KI) and visual tasks (VI).

**Feature**	***p*-value**	**KI Median ± MAD**	**VI Median ± MAD**
REC	**0.0467**	0.2699 ± 0.0152	0.2610 ± 0.0123
DET	**0.0372**	0.4359 ± 0.0199	0.4184 ± 0.0209
LAM	0.1127	2.3812 ± 0.0450	2.3608 ± 0.0456
ENTR	**0.0257**	0.5030 ± 0.0288	0.4814 ± 0.0295

**Table 3:** Statistical comparison between BI and GI.

**Feature**	***p*-value**	**GI Median ± MAD**	**BI Median ± MAD**
REC	**0.0300**	0.2596 ± 0.0103	0.2722 ± 0.0185
DET	**0.0448**	0.4197± 0.0169	0.4372 ± 0.0244
LAM	**0.0106**	2.3526 ± 0.0393	2.3881 ± 0.0546
ENTR	**0.0339**	0.4820 ± 0.0248	0.5040 ± 0.0318

**Table 4:** Statistical comparison between BI and GI considering only the easy task.

**Feature**	***p*-value**	**GI Median ± MAD**	**BI Median ± MAD**
REC	0.4813	0.2642 ± 0.0121	0.2691 ± 0.0160
DET	0.6648	0.4295 ± 0.0188	0.4275± 0.0159
LAM	0.2786	2.3722 ± 0.0454	2.3822± 0.0490
ENTR	0.6648	0.4935 ± 0.0198	0.4950± 0.0249

**Table 5:** Statistical comparison between BI and GI considering only the difficult task.

**Feature**	***p*-value**	**GI Median ± MAD**	**BI Median ± MAD**
REC	0.0655	0.2570 ± 0.0072	0.2734 ± 0.0186
DET	**0.0450**	0.4161 ± 0.0133	0.4440 ± 0.0281
LAM	**0.0264**	2.3457 ± 0.0358	2.4065 ± 0.0802
ENTR	**0.0422**	0.4767 ± 0.0212	0.5180 ± 0.0359

### 3.1. Neuropsychological Results

The scores obtained in the STAI-Y and PANAS questionnaires before and after the experimental session were compared by means of separate Repeated measures ANOVAs, with Groups (GI, BI) as between subject factor and TASK (Pre, Post) as within subject factor. No significant effects were found, thus indicating that the two groups had similar anxiety levels and mood. The experimental procedure did not induce any anxiety or negative affect that could impair the performance, e.g., an attention decrease.

### 3.2. Task Evaluation

The self-assessment (SA) scores (imagery easiness/difficulty) assigned to each task by good and bad imagers were compared by means of Mann–Whitney *U*-tests. No significant differences were found. Moreover, analysis of correlation (Spearmean) between SA and chronometry scores did not reveal any significant association between the two measures.

## 4. Discussion

The importance of MI has been well-documented in several domains such as medicine, education, training, or consumer behavior theory (MacInnis and Price, 1987). In this study, we propose a novel analysis for a more objective measurement of the MI ability in humans. Specifically, we analyzed the eye gaze dynamics to investigate the mental process underpinning imagination tasks. Indeed, the eye behavior can provide a valuable measure of an inner brain activity that cannot explicitly be analyzed, but whose dynamics can be reconstructed starting from its outcomes (i.e., the eye-gaze). Particularly, we have described the evolution of the imagery process through a set of complexity measures extracted from the phase space trajectory recurrences (Marwan et al., 2002).

Statistical results emphasized how the computed recurrence features, i.e., recurrence rate (*REC*), determinism (*DET*), laminarity (*LAM*), and entropy (*ENTR*), were able to significantly discriminate between GI and BI groups (see p-values in the Table 3), showing also how the complexity of the mental process changes between different levels of MI ability. Specifically, these results seem to indicate that BIs show a more complex mental process than the GIs, which instead shows a more predictable and ordered activity. In fact, the median of all metrics, i.e., *REC*, *DET*, *LAM*, and *ENTR*, was significantly higher in the BI group than in the GI group, especially when the imagery tasks became more difficult and a good imagery ability was increasingly necessary (Table 5). Moreover, the current results also show that while chronometric evaluation per se does not allow discriminating between visual and kinaesthetic modalities (Collet et al., 2011; Moran et al., 2012; Williams et al., 2015), the nonlinear dynamics of eye movements revealed also differences between the two imagery modalities.

Previous studies have described imagery as a processing mode in which multi-sensory information is represented in a gestalt form in working memory (MacInnis and Price, 1987), and have demonstrated how the dimensional complexity of imagery was consistently higher than perception (Schupp et al., 1994). Our results underlined the relationship between imagery and its generation components such as visual memories for recognition, or waking visual imagery (Farah, 1984). In fact, starting from the evidence that eye movements during visual imagery are related to what has been seen (Brandt and Stark, 1997), we showed that the nonlinear indexes of eye pattern significantly change according to the quality of the imagery performance and therefore this suggests that they contain information on the process of imagery. The higher REC and DET found in the BI group would likely reflect their bad working memory ability, which requires the recurrent fixation of the buttons on the screen to mentally reproduce the motor sequence (Gurtner et al., 2019). On the other hand, higher RQA parameters in the kinaesthetic modality than in the visual one can indicate facilitation for visual-motor imagery when visuospatial working memory is required. In addition, we enriched previous findings in the literature (Schupp et al., 1994), showing that complexity analysis of the eye behavior could provide a robust and accurate description of the imagery process. Indeed, it is worthwhile noting that nonlinear eye-gaze information is more informative than standard indexes such as time of fixation and pupil dilation, which did not provide any significant results. Furthermore, the accuracy and efficiency of the computed descriptors showed how the difficult tasks reached more evident results probably due to the required involved mental resources.

Of note, our non-linear indexes are statistically tested on groups defined based on the CM values. Therefore, we used a partially-subjective measure. This is a general limitation when validating physiological markers to infer the psychophysiological state. Indeed, a ground-truth and a comprehensive validity assessment protocol for validation of physiological signals are not possible to be performed.

In conclusion, imagery processing affects a multitude of cognitive, physiological, and behavioral phenomena in many domains such as learning, problem-solving, and consumer experiences. Our work opens new windows for a better understanding of how motor imagery performance can influence these phenomena.

Future endeavors will be directed toward the comparison of our non-linear indexes with results derived from the event-related desynchronization/synchronization analyses in an EEG-based brain-computer-interface scenario.

## Data Availability Statement

The datasets generated for this study are available on request to the corresponding author.

## Ethics Statement

This study was carried out in accordance with the recommendations of Helsinki declaration with written informed consent from all subjects. All subjects gave written informed consent in accordance with the Declaration of Helsinki. The protocol was approved by the Bioethics committee of the University of Pisa, N. 3/2019.

## Author Contributions

AL, LS, and FD contributed to the design and implementation of the research. SD, LS, and AG contributed to the analysis of the results. AL, LS, FD, SD, ES, and AG equally contributed to the writing of the manuscript. All authors have approved the work for publication.

### Conflict of Interest

The authors declare that the research was conducted in the absence of any commercial or financial relationships that could be construed as a potential conflict of interest.
